# Oncolytic vaccinia virus immunotherapy antagonizes image-guided radiotherapy in mouse mammary tumor models

**DOI:** 10.1371/journal.pone.0298437

**Published:** 2024-03-18

**Authors:** Brittany A. Umer, Ryan S. Noyce, Quinten Kieser, Nicole A. Favis, Mira M. Shenouda, Kim J. Rans, Jackie Middleton, Mary M. Hitt, David H. Evans

**Affiliations:** 1 Department of Medical Microbiology and Immunology, University of Alberta, Edmonton, Alberta, Canada; 2 Li Ka Shing Institute for Virology, University of Alberta, Edmonton, Alberta, Canada; 3 Department of Oncology, University of Alberta, Edmonton, Alberta, Canada; University of Surrey, UNITED KINGDOM

## Abstract

Ionizing radiation (IR) and oncolytic viruses are both used to treat cancer, and the effectiveness of both agents depends upon stimulating an immune response against the tumor. In this study we tested whether combining image guided ionizing radiation (IG-IR) with an oncolytic vaccinia virus (VACV) could yield a better therapeutic response than either treatment alone. ΔF4LΔJ2R VACV grew well on irradiated human and mouse breast cancer cells, and the virus can be combined with 4 or 8 Gy of IR to kill cells in an additive or weakly synergistic manner. To test efficacy *in vivo* we used immune competent mice bearing orthotopic TUBO mammary tumors. IG-IR worked well with 10 Gy producing 80% complete responses, but this was halved when the tumors were treated with VACV starting 2 days after IG-IR. VACV monotherapy was ineffective in this model. The antagonism was time dependent as waiting for 21 days after IG-IR eliminated the inhibitory effect but without yielding any further benefits over IR alone. In irradiated tumors, VACV replication was also lower, suggesting that irradiation created an environment that did not support infection as well *in vivo* as *in vitro*. A study of how four different treatment regimens affected the immune composition of the tumor microenvironment showed that treating irradiated tumors with VACV altered the immunological profiles in tumors exposed to IR or VACV alone. We detected more PD-1 and PD-L1 expression in tumors exposed to IR+VACV but adding an αPD-1 antibody to the protocol did not change the way VACV interferes with IG-IR therapy. VACV encodes many immunosuppressive gene products that may interfere with the ability of radiotherapy to induce an effective anti-tumor immune response through the release of danger-associated molecular patterns. These data suggest that infecting irradiated tumors with VACV, too soon after exposure, may interfere in the innate and linked adaptive immune responses that are triggered by radiotherapy to achieve a beneficial impact.

## Introduction

Immunotherapy mobilizes a patient’s immune system to target and kill cancerous cells and has advanced rapidly in the last decade. Much success has been achieved using the approach to treat hematological malignancies [1, 2], although it has not achieved the same success at treating solid tumors, such as breast cancers [3]. As research in the field progresses, there has been a growing interest in combining immunotherapies with other treatments, such as chemotherapeutic drugs [4] and radiation [5], to broaden the utility of the approach as well as to achieve a therapeutic effect superior to single-agent treatments alone.

Oncolytic viruses promote another form of immunotherapy in which a virus is used to infect and kill tumor cells, while also stimulating anti-tumor immunity [6, 7]. To date, several oncolytic viruses have received approvals as therapeutics in different jurisdictions to treat cancers. These include an adenovirus for treating nasopharyngeal carcinoma (H101, China) and herpes simplex viruses that are approved for use in treating glioblastoma (Japan) and for melanoma (T-VEC, USA) [7]. How the therapeutic benefits of oncolytic virus therapies in humans might be enhanced in combination with other forms of immunotherapy is a subject of ongoing investigation [8, 9].

Our laboratory studies oncolytic vaccinia virus (VACV), a large DNA virus once widely used to vaccinate against smallpox [10]. By deleting viral genes encoding components of the nucleotide metabolism machinery, F4L (the small subunit of ribonucleotide reductase) and J2R (thymidine kinase), a ΔF4LΔJ2R VACV strain is rendered dependent on the host cell to produce dNTPs for virus replication [11, 12]. These deletions in F4L and J2R restrict virus replication to growing tumors and improve safety, while maintaining therapeutic efficacy and promoting anti-tumor immunity in bladder cancer models [13]. However, despite this success in treating orthotopic mouse and rat models of bladder cancer, ΔF4LΔJ2R VACV was not therapeutically beneficial when tested in mouse breast tumor models [14].

Previous pre-clinical studies have shown that different oncolytic viruses can sometimes synergize with ionizing radiation (IR) therapy to produce improved therapeutic responses [15]. Various mechanisms are likely responsible. For example, IR can upregulate cellular ribonucleotide reductase activity and this appears to increase the titers and oncolytic activity of a herpes simplex virus (HSV) [16]. Some recombinant VACV also yield improved outcomes when virus treatments are combined with different forms of radiation [17–21]. We recently reported a striking example of the benefits of combining ΔF4LΔJ2R VACV with IR to treat aggressive orthotopic glioblastoma tumors in immune-competent mice. This study showed that 10 Gy of IR combined with ΔF4LΔJ2R VACV improved the long-term survival from 13% and 21% with virus or radiation alone, respectively, to 67% when radiation was followed by 10^7^ PFU of virus [22]. This showed that an improved therapeutic outcome could be achieved by combining ΔF4LΔJ2R VACV with IR and it raises the question of whether the effect can be obtained in other pre-clinical models of cancer, targeting tumor types that have proven resistant to oncolytic virus treatment.

One factor that complicates the interpretation of some earlier studies, is that the way the radiation was delivered rarely replicated the way tumors are treated in humans. Current methods use image-guided radiation to ensure that most of the dose is distributed in a region defined by the tumor boundaries [22, 23]. In our glioblastoma study [22], and herein, we assessed treatment outcomes when VACV was combined with image-guided ionizing radiation (IG-IR) delivered using a small animal radiation research platform (SARRP). IG-IR combines tumor mapping with radiation delivery, allowing the operator to deliver a precise dose of radiation to the tumor bed with high spatial accuracy. This greatly reduces off-target damage to healthy tissues like nearby lymphoid organs. We speculated that one reason for the lack of efficacy when ΔF4LΔJ2R VACV is used to treat mammary tumors [14] may be because the virus alone is incapable of debulking these often aggressive tumors. The current project tests whether an initial dose of IG-IR can be used to reduce the bulk of the tumor mass, leaving the ΔF4LΔJ2R VACV to infect and kill any tumor cells that remain alive on the tumor periphery.

## Results

### IG-IR and ΔF4LΔJ2R VACV synergize to kill breast cancer cells *in vitro*

**Fig 1:**
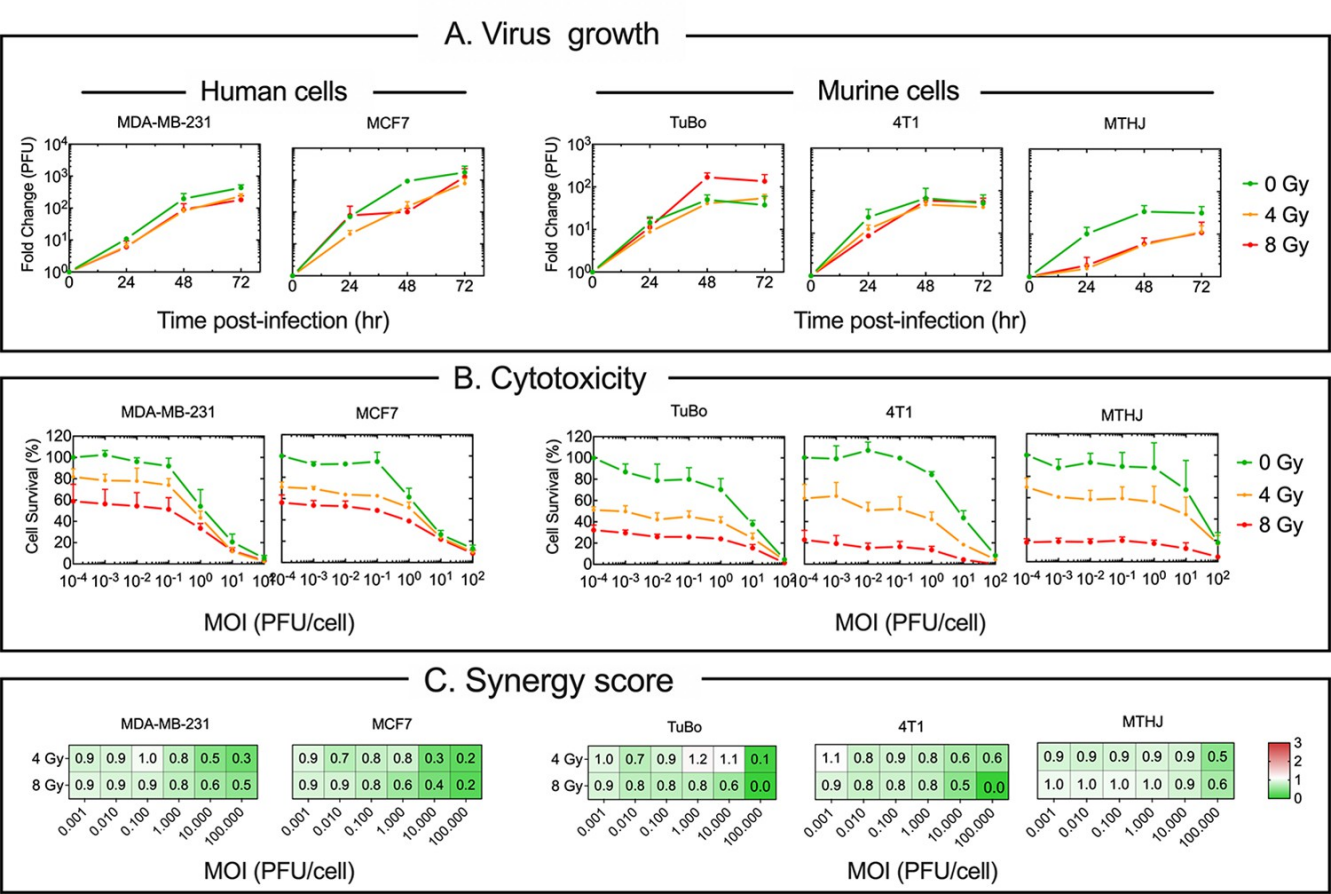
Radiation and ΔF4LΔJ2R VACV can combine synergistically to kill breast cancer cells *in vitro*. Panel A. Virus growth in cells treated with 0, 4, or 8 Gy IR. Cells were infected with virus at MOI = 0.03 PFU/cell, 24 hr post-irradiation, and the yield determined by plaque assay on BSC-40 cells. Panel B. Cell killing. Cells were exposed to 0, 4, or 8 Gy IR and then infected (or mock infected) 24 hr later with VACV at the indicated MOIs. Three days after infecting the cells, a dye reduction assay was used to measure viability. Panel C. Heat-maps showing CompuSyn combination index (CI) values. Green indicates synergistic behavior (CI<0.9), white additive effects (CI = 0.9–1.1), and red is antagonistic (CI>1.1) The CI’s were calculated using average viability values shown in panel B. The error bars in panels A and B show ± SEM of three experimental replicates.

We first tested whether radiation had any effects on VACV growth in human and mouse breast cancer cell lines. The cells were exposed to 4 or 8 Gy of gamma radiation from a cesium source, or mock-irradiated as a control. Twenty-four hours later the cells were infected with virus at a low multiplicity of infection (MOI; 0.03 PFU/cell) and the yield of virus determined at subsequent time points by plaque assay on BSC-40 cells. The virus yield was unaffected by 4 or 8 Gy IR in most of the cell lines tested (Fig 1A). We next assessed the cytotoxicity caused by combining radiation plus ΔF4LΔJ2R VACV using the same panel of cell lines. Cells were irradiated, or mock irradiated, and then infected 24 hr later with a MOI of virus varying from 0 to 100 PFU/cell (Fig 1B). Infecting irradiated cells with VACV further enhanced cell death in all the cells tested, most clearly at the higher MOI’s (Fig 1B). This showed that VACV does not impair the ability of radiation to cause cell death *in vitro*.

To quantify the drug-like interactions between the two treatments, we used CompuSyn Drug Synergy analysis software. This analysis takes data from viability assays when two drugs are combined and computes a combination index (CI) value to determine how drugs are interacting [24, 25]. Briefly, CI<1 is considered synergistic, CI = 1–1.1 is considered additive, and CI>1.1 is antagonistic. The CI values showed that for the most part combinations of IR and ΔF4LΔJ2R VACV were almost exclusively synergistic (Fig 1C). The singular exception being in the TUBO cell line at 4 Gy of radiation with MOI = 1, where CI = 1.2 points to an additive or slightly antagonistic effect in play. Overall, the increased cytotoxicity that is seen when one combines IR and virus suggested that a combination of two treatments would be more beneficial than either treatment alone. These results led us to evaluate the effects of combining these therapies *in vivo*.

### VACV therapy antagonizes IG-IR *in vivo*

**Fig 2:**
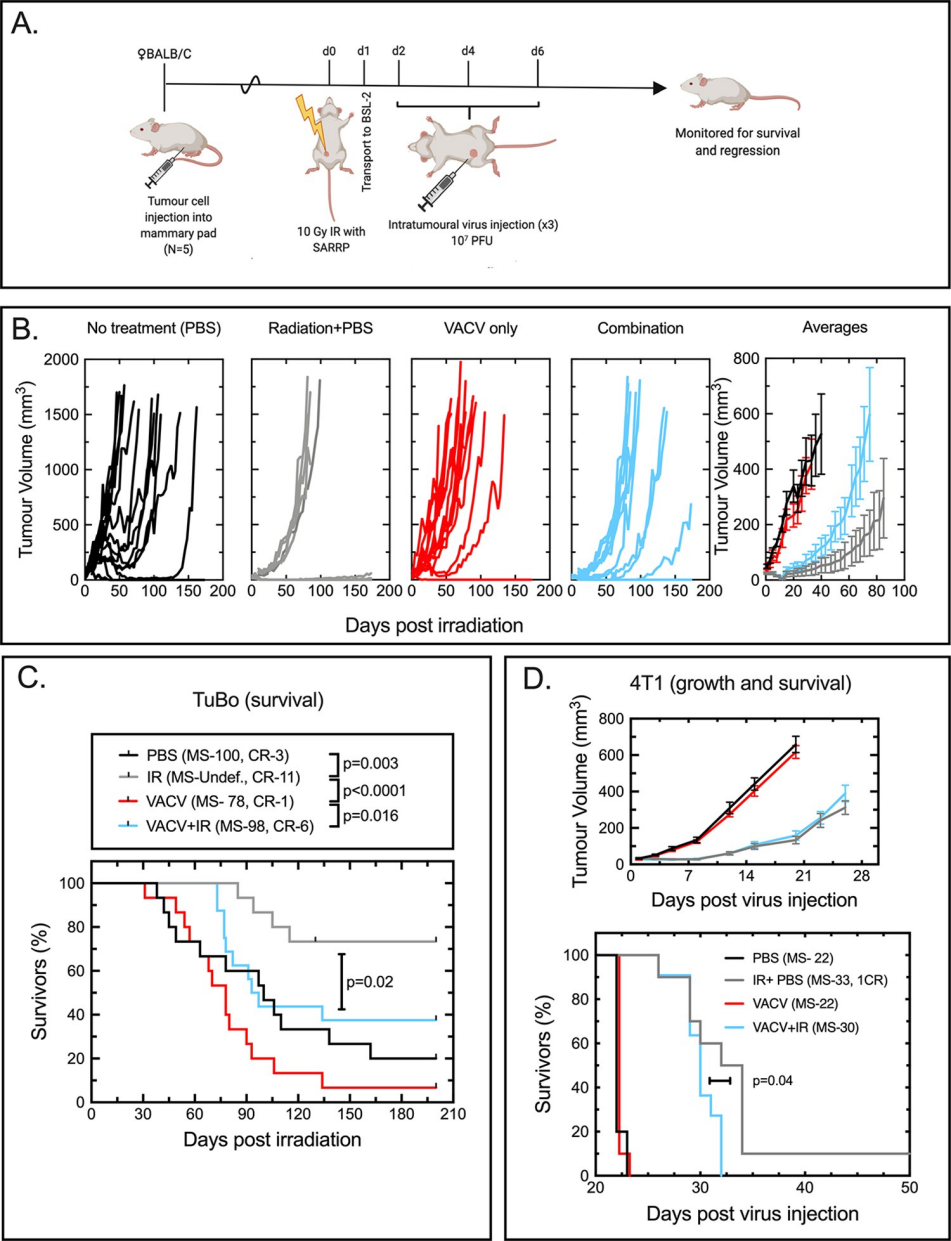
The therapeutic benefits of IG-IR therapy are either antagonized, or unresponsive, to ΔF4LΔJ2R VACV virotherapy. Panel A. Protocol and timeline used to test the effects of combining IG-IR with VACV. Image created using BioRender.com. Panel B. TUBO tumor growth in each mouse measured twice per week after treatment; 10 Gy IR followed by injections of PBS; VACV alone; or 10 Gy IR followed by ΔF4LΔJ2R VACV. The fifth plot shows the average tumor volumes in each treatment group. The plots terminate when the first mouse in each group reached endpoint. Error bars denote +/- SEM. Panel C. Kaplan-Meier survival plot with p values calculated using a Mantel-Cox log-rank test. The median survival time (MS) and number of complete responses (CR) are also reported. Each TUBO treatment group comprised 15 animals (5 mice per experiment, replicated three times). Panel D. 4T1 orthotopic and metastatic tumor model. The same experimental regimen shown in Panel A was used to follow tumor development and construct a survival plot. The p value was calculated using a Mantel-Cox log-rank test and only comparisons indicating a significant difference between survival curves are show. Each 4T1 treatment group comprised 10 or 11 mice (5 or 6 mice per experiment, replicated twice).

To test a combination therapy regimen *in vivo*, we injected 6-8-week-old female BALB/C mice with TUBO cells in the mammary fat pad (Fig 2A). Once tumors were palpable (~8 days post injection), the mice were anaesthetized, and a SARRP irradiator was used to deliver 10 Gy of IG-IR to each tumor isocenter. The next day the mice were transported to the BSL-2 vivarium, where they were allowed to acclimatize overnight. Two days post-irradiation, each tumor was injected with 10^7^ PFU of ΔF4LΔJ2R VACV (or PBS as a mock control), and this was repeated twice more at 48 hr intervals. Each animal’s weight and tumor dimensions were measured twice weekly until reaching an endpoint determined by tumor size and health score. Five mice were used per group per experimental repeat, and the experiment was performed three times for a total of 15 mice per group. It should be noted that the SARRP is located at a site where replicating viruses are not permitted by biosafety policy. This precluded testing an alternative treatment order employing virus followed by IG-IR.

Despite the mostly synergistic effects seen *in vitro*, we saw that ΔF4LΔJ2R VACV treatment antagonized IG-IR in this orthotopic and syngeneic animal tumor model (Fig 2). When comparing the rates of tumor growth, there was no difference between TUBO tumors injected with PBS and those treated with VACV (Fig 2B). By itself, IR alone is effective and delayed tumor growth in many animals. However, ΔF4LΔJ2R VACV decreased the effectiveness of IR, as tumors grew more rapidly when IR was followed by VACV injections. Survival was also significantly (p = 0.02) decreased in the IR plus VACV group relative to IR alone (Fig 2C). We observed 11/15 complete responses (CR) in radiation-treated animals and a median survival time >200 days compared to IR plus VACV where 6/15 mice exhibited CR with a median survival of 98 days. A general impression would be that compared to VACV alone (1 CR) and IG-IR (11 CR), VACV plus IR yields an outcome in-between the two single modes of treatment (6 CR).

To see if this antagonism was restricted to the TUBO tumor model, we also examined whether the same results would be obtained using a 4T1 tumor model. This orthotopic and syngeneic mammary tumor is more aggressive than TUBO tumors and also responds poorly to VACV therapy [7]. Long term cures are rarely reported. Using the same treatment regimen as shown in Fig 2A, 4T1 tumors were established in BALB/C mice and treated as described above. As with the TUBO model, IG-IR alone induced the best therapeutic response, extending the median survival by 11 days relative to PBS alone (Fig 2D). ΔF4LΔJ2R VACV treatment provided no improvement over PBS alone. We did see a very small but still statistically significant decrease in median survival in mice treated with IR plus VACV compared with mice treated with radiation alone (30 versus 33 days, p = 0.04; Fig 2D), suggesting that in both breast tumor models, combining IR with ΔF4LΔJ2R VACV may antagonize IR’s therapeutic benefits. However, the overall survival was not much affected (0 CR in the VACV+IR cohort versus 1 CR in the IR+PBS group), providing no evidence to suggest that combining therapies yields superior outcomes.

### Delaying VACV treatment prevents virus interference in the response to IG-IR

**Fig 3:**
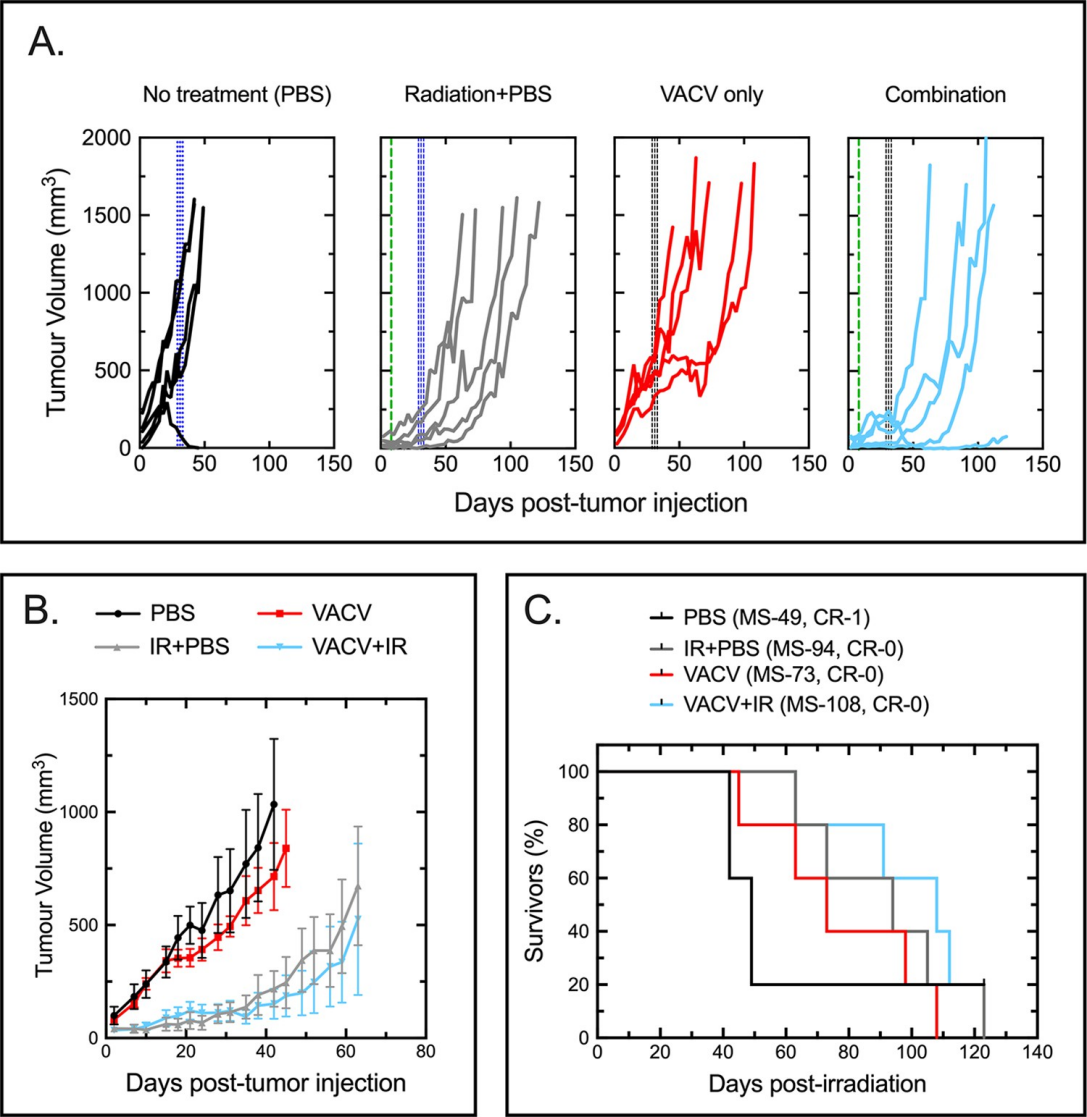
Delaying virus administration minimizes virus interference in IR therapy but does not improve survival times. In this study the first of three doses of virus (black dashed lines) or PBS (blue dashed lines) were administered 21 days after IR therapy (green dashed lines). Panel A. Tumor growth in each mouse treated using the indicated regimen. Panel B. Averages of the tumor volumes from panel A. Each plot was terminated when the first mouse in each group reached endpoint. Error bars show +/- SEM. Panel C. Kaplan-Meier survival plot. No significant difference in median survival were found between any of the treatment groups. Each treatment group comprised 5 mice.

We observed that when the VACV injections were begun 48 hours after irradiating the TUBO tumors, the combination seemed to partially control tumor growth for about three weeks after virus treatment (Fig 2B). Thereafter the tumor growth resumed and in the VACV+IR cohort it overtook the rate of growth seen when mice were treated with just radiation. This led us to wonder whether this might be a better time to exploit the oncolytic properties of ΔF4LΔJ2R VACV and perhaps also avoid the antagonism we saw when the virus was introduced immediately after IR therapy. This led us to test whether delaying virus treatment until 21 days after IG-IR treatment might improve the overall response to combination therapy (Fig 3).

Delaying the start of the virus injections for three weeks following IG-IR treatment did change the course of the experiment. Perhaps the most notable effect was that the exponential increase in tumor size that begins to be seen in many animals two-to-three weeks after irradiation, is delayed and in some cases transiently reversed by ΔF4LΔJ2R VACV treatment (Fig 3A and 3B). This pause in tumor growth was ultimately associated with a small increase in the median survival in the IR plus virus group compared to the IR only cohort. Although the difference between the two groups was not statistically significant (MS = 94 versus 108 days, p = 0.66; Fig 3C), delaying administration of the virus meant it no longer antagonized the anti-tumor response initiated by IR. Nevertheless, this altered protocol still did not improve the overall outcome, as a combination of IG-IR and virus therapy didn’t ultimately enhance survival relative to the radiation alone treatment group (Fig 3C).

### VACV changes the tumor and splenic immune cell microenvironment created by IG-IR

**Fig 4:**
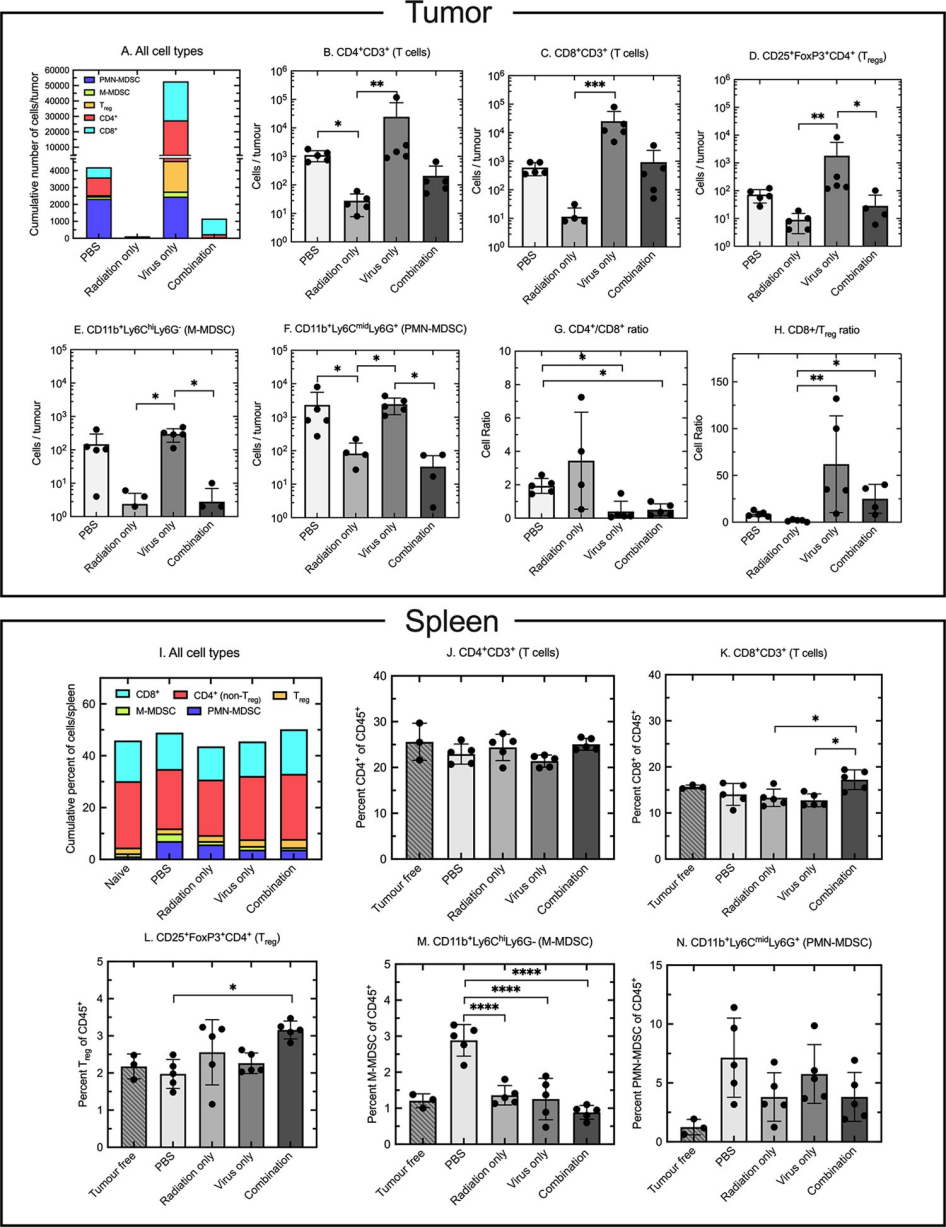
Combining radiation and VACV therapy changes the TUBO tumor and splenic immune cell microenvironment. Tumors were established and mice treated as in Fig 2A, then the tissues retrieved and processed one week after the last virus (or mock virus) treatment. Tumors and spleens are analyzed in the upper (A-H) and lower (I-N) panels, respectively. Panel A, cumulative average numbers of the indicated cell types, per tumor, in each treatment group. Panels B-F, total numbers of (B) CD4^+^ T cells, (C) CD8^+^ T cells, (D) T_reg_, (E) M-MDSC cells, and (F) PMN-MDSC cells. Panels G-H show the CD4^+^/CD8^+^ and CD8^+^/T_reg_ cell ratios. Panel I shows the immune cells recovered from spleens of mice harboring TUBO tumors. The remaining portion were non-MDSC CD11b^+^ and non-CD3^+^ non-CD11b^+^ cell types. Naïve tumor-free mice are shown for comparison. Panels J-N shows (J) CD4^+^ cells, (K) CD8^+^ cells, (L) T_reg_ cells, (M) M-MDSC cells, and (N) PMN-MDSC cells expressed as a percentage of CD45^+^ cells. These averages were calculated from 5 mice per treatment group and 3 mice in the naïve (spleens only) group. Each data point represents a single mouse. Error bars depict the 95% CI from the mean. P values were calculated using ANOVA or Kruskal-Wallis testing if the data was shown to be parametric or non-parametric, respectively. P-values are coded as *p<0.05, **p<0.01, and ****p<0.005.

Radiation kills cancer cells and in doing so can induce both immunogenic and immunosuppressive responses towards the tumor cells and in the surrounding tumor microenvironment (TME) [26–28]. Oncolytic viruses also provoke immune responses that, although principally directed against the infecting agent, can also induce or enhance immune responses towards infected tumor cells. We hypothesized that infecting tumors with ΔF4LΔJ2R VACV, soon after irradiating the cells, somehow altered the immune cell composition in the TME in a way that interfered with the advantageous effects of radiation. To test this hypothesis, we used the original treatment protocols (Fig 2A) and elected to assess the immune cell composition in TUBO tumors one week after administering the final (third) dose of virus. This is about 2 weeks after the one-time treatment with IG-IR was delivered. Flow cytometry was used to identify Immune cells in tumor tissues and spleens recovered from treated animals (Fig 4).

Perhaps the most informative data were obtained from an analysis of the numbers and types of tumor-infiltrating lymphocytes (TILs) and immunosuppressive myeloid cells harvested at this time point. The composition was dominated by PMN-MDSC and CD4^+^ cells in the PBS-treated controls (Fig 4A). Virus treatment increased these numbers ~10-fold, through a considerable expansion of the CD4^+^, CD8^+^, and T_reg_ populations. The impact of radiation was striking as few TILs of any type could be isolated from the TME treated with IR alone: on average only a few hundred cells per tumor. Mice subjected to both treatments showed an increase in numbers relative to IG-IR alone, however infiltration was still limited with an average of only ~1,100 profiled immune cells recovered per tumor, with CD8^+^ and some CD4^+^ cells predominating (Fig 4A).

These differences in immune cell composition are further illustrated using a side-by-side comparison of how different treatments affected the numbers of different cell types (Fig 4B–4H). VACV treatment significantly increased the numbers of all cell types analyzed (CD4^+^, CD8^+^, T_reg_, M-MDSC, and PMN-MDSC) relative to tumors treated with IR alone (Fig 4B–4F). This undoubtably reflects the effects of IG-IR therapy which considerably depleted the numbers of all immune cell types. Where a combination of IR plus virus treatment was employed, two effects were seen. First it seemed that the addition of virus treatment had little effect on the T_reg_, M-MDSC and PMN-MDSC cell populations, which persisted at levels comparable to those seen in tumors treated with IR alone (Fig 4D–4F). At the same time, the CD4^+^ and CD8^+^ cell counts in tumors treated with both agents fell in-between the counts in tumors treated with single therapies. Paradoxically, these data show that combination therapy resulted in what are generally viewed as positive changes to the tumor immune cell microenvironment. That is, we found increased numbers of CD8^+^ T cells relative to the T_reg_ populations (Fig 4H) and immunosuppressive myeloid cell populations were reduced to levels seen in IR treated tumors (Fig 4E and 4F). In the combination therapy group, CD4^+^ and CD8^+^ cell numbers were higher compared to radiation treatment alone, more closely resembling CD4^+^ and CD8^+^ cell numbers in untreated (PBS) tumors (Fig 4B and 4C). However, the differences were not statistically significant.

We also performed an analysis of cells recovered from the non-irradiated spleens of these mice. This showed that the overall average immune cell composition was not greatly affected by the different treatments (Fig 4I). However, some differences were seen when comparing individual immune cell subtypes (Fig 4J–4N). While there was no change in the percentage of splenic CD4^+^ T cells, there was a small increase in the proportion of CD8^+^ T cells after combination therapy relative to tumors treated with IR or virus alone (Fig 4J and 4K). This was associated with a small increase in the percentage of T_reg_ cells in the combination group relative to the PBS-treated controls (Fig 4L), with the result that the ratio of CD8^+^ T cells to T_reg_ cells remained little changed across the three treatment regimens. The M-MDSC cell content declined significantly in all the treated animals compared to PBS-treated tumors (Fig 4M), but with PMN-MDSC cells there were no striking differences between IR, virus alone, or combination therapy.

Immune analysis of 4T1 tumors showed an interesting pattern in that by all the measured criteria, combining the two treatments produced outcomes almost identical to radiation alone (S1 Fig). The highest levels of CD4^+^, CD8^+^, T_reg_, and M-MDSC cells were detected in tumors treated with virus alone, significantly more than were detected in irradiated tumors but not much altered compared to the PBS-treated controls. Radiation plus virus depleted the tumors of all TIL populations and suppressive myeloid cells down to levels essentially identical to those detected with radiation alone (S1 Fig, upper panel). A very similar response was detected in the spleens. That is, virus alone looked much like the PBS-treated controls and virus plus irradiation looked much like radiation alone. This is fully consistent with the survival curves where the PBS and VACV plots are nearly identical as are IG-IR and IG-IR plus VACV (Fig 2D).

In parallel with these analyses we also examined effects on virus yields. Irradiation decreased the yields of virus in both irradiated TuBo and 4T1 tumors compared to non-irradiated tumors (S2 Fig) while no viruses were detected in any of the other organs tested in either treatment group (spleens, draining lymph nodes, heart, or liver). Radiation damage compromises the tumor microvasculature in ways not reflected in vitro, and these data suggest that irradiating tumors creates an environment that more poorly supports VACV replication *in vivo*.

### Treating TUBO tumors *in vivo* with IG-IR and/or VACV generates T-cells with specificity towards viral and tumor epitopes

**Fig 5:**
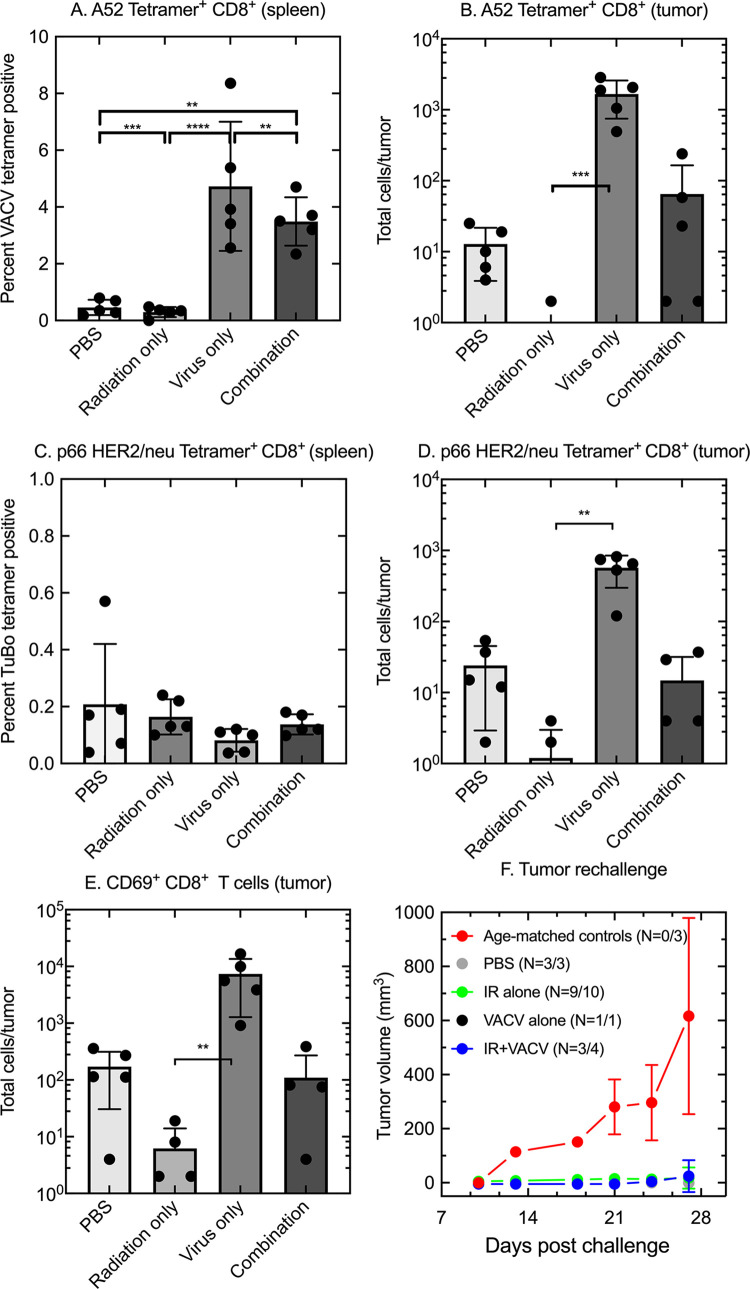
CD8^+^ T-cell specificity. TUBO tumors were established and mice treated as shown in Fig 2A. Panels A-E. Lymphocytes were isolated from the tumors and spleens and then harvested one week after the last of three virus or PBS treatments (N = 4–5 mice). Panel A. Percentage of splenic CD8^+^ T cells detected by a tetramer bearing a VACV A52_75-83_ peptide. Panel B. Total number of tumor infiltrating VACV A52_75-83_ tetramer positive CD8^+^ T cells. Panel C. Percentage of TUBO p66-HER2/*neu* tetramer positive CD8^+^ T cells from spleens. Panel D. Total number of tumor-infiltrating p66-HER2/*neu* tetramer positive CD8^+^ T cells. Panel E. Total number of CD8^+^ T cells detected in TUBO tumors bearing the activation marker CD69^+^. F. TUBO tumor re-challenge study in mice that had cleared a primary tumor (“cured” mice from experiment shown in Fig 2). Mice were implanted with fresh TUBO cells in the mammary fat pad opposite the site of the original tumor, and tumor growth measured twice weekly for a month. “N” is the number of animals that were tumor free at the end of the trial (numerator) and the number of survivors of each treatment type that were challenged (denominator). Error bars depict 95% CI from the mean, where *p<0.05, **p<0.01, ***p<0.005, and ****p<0.001.

The CD8^+^ T cells detected in spleens and TUBO tumors were further characterized with regards to the specificity of the T cell receptors. To do this we prepared H-2k^d^-restricted tetramers designed to detect T-cell receptors targeting virus or tumor peptide antigens. These tetramers used the immunodominant HER2/*neu* p66 peptide epitope expressed by TUBO cells [29] or the A52_75-83_ peptide epitope encoded by the VACV A52R gene [30]. As anticipated, VACV treatment alone or combined with IR induced a significant increase in the percent of CD8^+^ T cells in the spleen recognizing the A52 epitope (Fig 5A). These cells were also recovered in significant numbers from the tumor site in mice treated with virus alone, but the number of VACV-directed CD8^+^ T cells retrieved from tumors exposed to the combination therapy was lower and approached the background seen in many of the uninfected (PBS) mice (Fig 5B). In contrast to the virus-targeted CD8^+^ T-cells, we did not see any differences in the spleens between the control and treatment groups with respect to a HER2/*neu*-directed T cell response (Fig 5C). Virus treatment did significantly increase the numbers of CD8^+^ T cells targeting the HER2/*neu* epitope in tumors whereas these cells were almost undetectable in tumors treated with IG-IR alone (Fig 5D). It was notable that the large difference in the ratio of VACV-to-TUBO specific T cells that are detected in the spleens in animals treated with virus alone (~80-fold) was reduced to ~3-fold among cells residing in the tumors. The stimulatory effect of virus on the number of TUBO-specific T cells was not replicated in tumors treated with both IR and virus, where the numbers of HER2/*neu*-directed T cells more closely resembled those detected in the PBS-treated control (Fig 5D). Finally, we also examined the levels of CD69 expression on T cells isolated from tumor bearing mice. CD69 is widely used as a marker of T cell activation [31]. In tumors, the numbers of activated CD69^+^ CD8^+^ T cells was highest in the virus treated group, and lowest in radiation-only treated tumors (Fig 5E). However, although the number of CD69^+^CD8^+^ cells varied greatly from treatment to treatment, the relative abundance closely paralleled the numbers of CD8^+^ cells (Fig 4C) as well as the numbers of virus- and TUBO-specific CD8^+^ T cells (Fig 5B and 5D). This suggested that there is little difference in the proportion of activation of CD8^+^ T cells recovered from TUBO tumors.

Whether these virus- and TUBO-specific CD8^+^ T cells could promote anti-tumor immunity was further assessed in tumor rechallenge experiments. The mice that had previously cleared a TUBO tumor by day 200 post-implantation (Fig 2C), were rechallenged with fresh tumor cells in the opposite mammary fat pad. Sixteen of the 18 animals tested in this study either immediately rejected the tumor or had cleared the tumor by four weeks after the secondary tumor rechallenge (Fig 5F). The two mice that failed to control the new tumors had been previously treated with IR only (1 of 10 tested survivors) or IR plus ΔF4LΔJ2R VACV (1 of 4). The numbers are few but do show that functional anti-tumor immunity could be achieved in all four treatment groups.

Collectively, these data suggest that CD8^+^ T cells targeting both viral and tumor antigens are present after combination therapy, and that the T cells at the tumor site are activated. These data strongly suggest that T cells at the tumor site are functional and likely prevent establishment of secondary tumors.

### Immune checkpoint expression is enhanced by combination therapy

**Fig 6:**
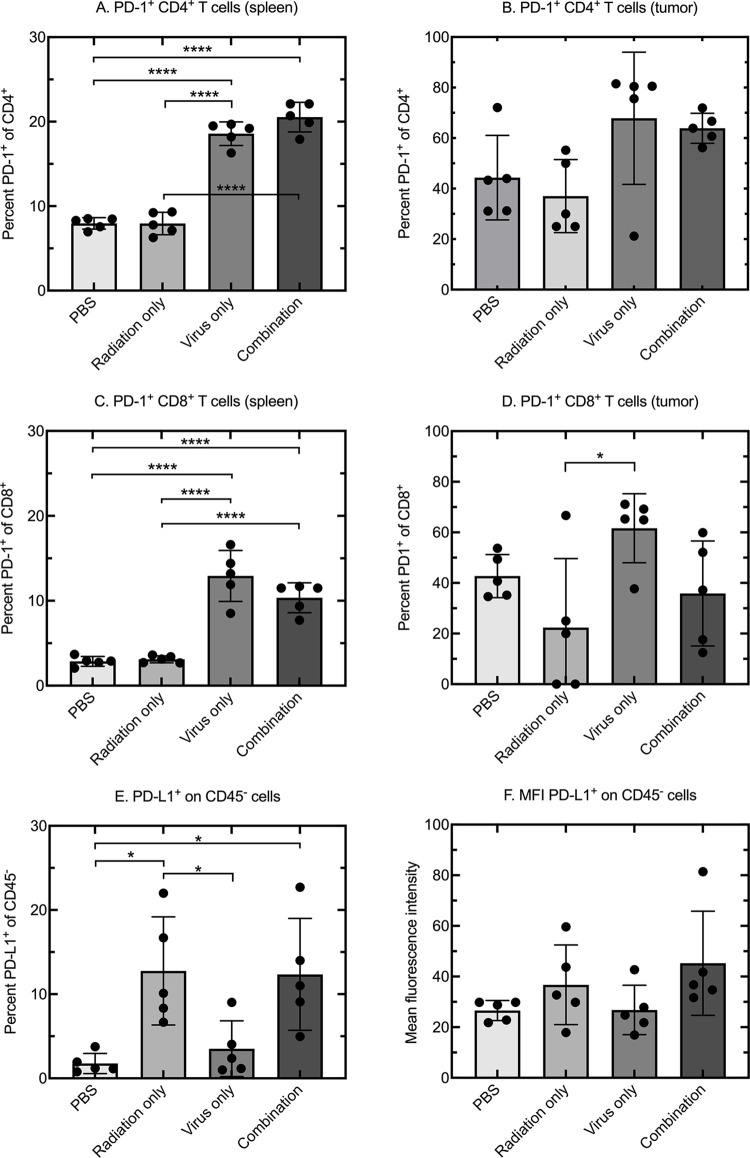
Combination therapy increases immune checkpoint expression in the tumor microenvironment. TILs were isolated from mice bearing TUBO tumors one week after the final virus treatment (or mock treatment) with a combination of IG-IR and/or VACV. The timing was otherwise as in Fig 2A. Panels A and B. Percentage of PD-1 positive CD4^+^ cells isolated from spleens and tumors, respectively. Panels C and D Percentage of PD-1 positive CD8^+^ cells isolated from spleens and tumors, respectively. Panels E and F. PD-L1 expression on CD45^-^ cells isolated from treated TUBO tumors, shown as either percent PD-L1^+^ or median fluorescence intensity (MFI), respectively. Each data points represents a different mouse. Error bars depict 95% CI from the mean; *p<0.05, **p<0.01 ***p<0.005 and ****p<0.001 assessed using one-way ANOVA where data were shown to be parametric or Kruskal-Wallis testing if data were non-parametric.

We next examined whether combining therapies had any impact on the negative checkpoint regulators of immune responses. We observed that PD-1 was selectively upregulated on the T cells recovered from the spleens of the virus-treated animals (Fig 6A and 6C). CD4^+^ T cells isolated from the spleens of mice treated with PBS or IG-IR expressed the lowest levels of PD-1 (<10%), whereas the CD4^+^ cells from mice exposed to virus treatment, with or without IR, exhibited a doubling of the proportion of cells expressing the marker (Fig 6A). A similar trend was observed in the cells isolated from the tumors combined with a ~2-fold increase across all groups in the proportion of CD4^+^ cells expressing the PD-1 marker compared to that in the spleen (Fig 6B). Similar to our analysis of CD4^+^ cells, we detected very low levels of PD-1 expression in splenic CD8^+^ cells from the PBS or IG-IR treatment groups and a significant (2-3-fold) increase in those from the VACV-treated animals (Fig 6C). The trend was less clear in the tumor-derived CD8^+^ cells although again a substantial increase in the proportion of PD-1 positivity was seen in virus-treatment groups. Relative to the splenic CD8^+^ cells, the highest proportion of CD8^+^PD-1^+^ cells (~60%) was detected in cells isolated from mice treated with VACV alone (Fig 6D).

PD-1 is a marker of T cell activation that renders the cell susceptible to exhaustion through engagement with its ligands. PD-1 suppresses T cell responses through binding to PD-L1 or PD-L2, and these ligands are expressed on many tumor cells [32, 33], as well as other cell types. We measured the levels of PD-L1 expression on CD45^-^ cells (non-immune cells) isolated from treated TUBO tumors. We found that radiation therapy, with or without virus treatment, increased the percentage of PD-L1^+^CD45^-^ cells in tumors relative to PBS treated tumors (Fig 6E). However, this increase in the proportion of PD-L1 positive cells was not associated with much increase in the relative level of expression on PD-LI positive cells (Fig 6F).

### Anti-PD-1 checkpoint therapy did not reverse the antagonism seen when IG-IR is combined with virotherapy

**Fig 7:**
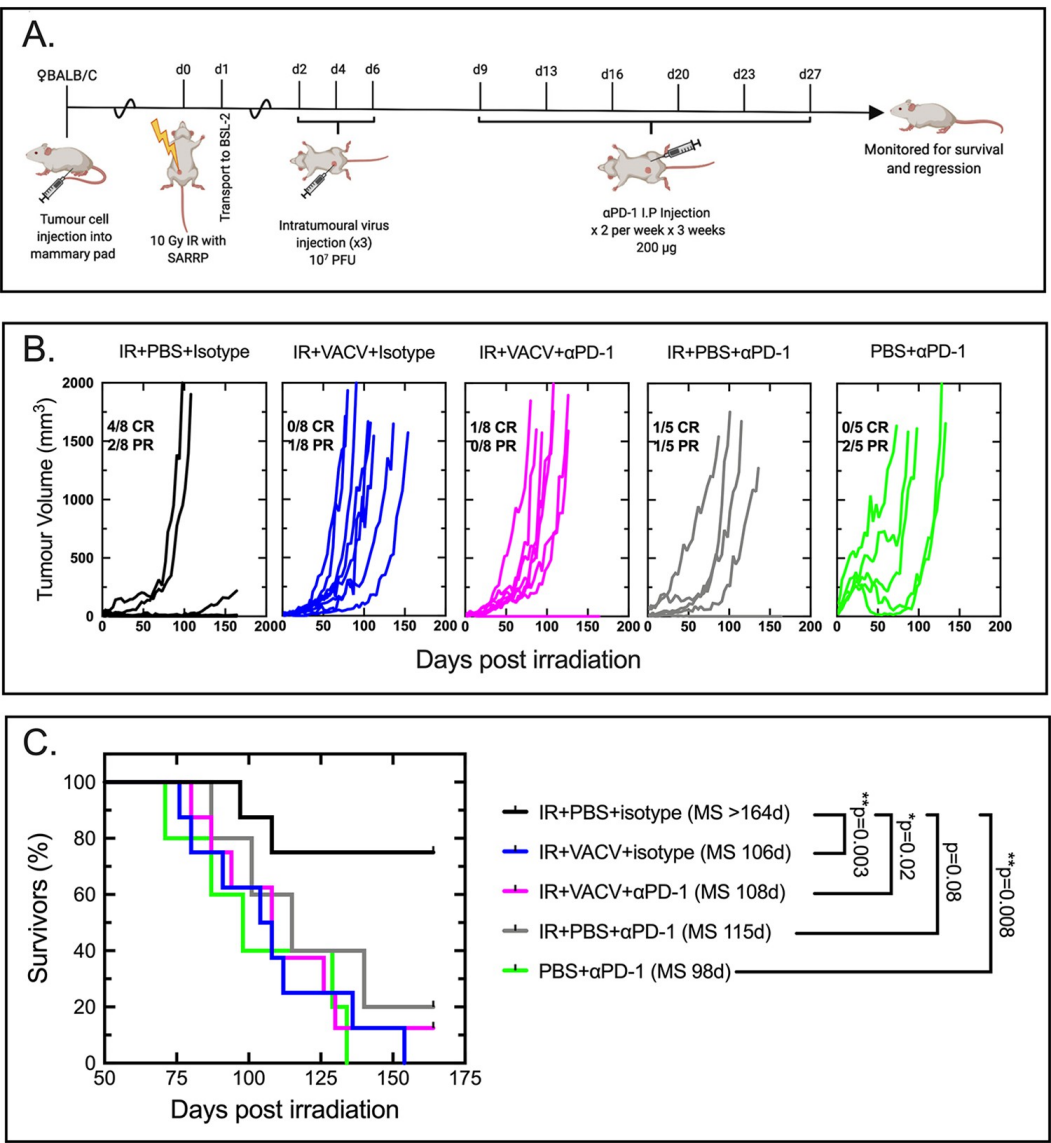
Anti-PD-1 checkpoint therapy does not reverse VACV antagonism of image-guided radiotherapy. Panel A. Experimental outline. Panel B. Tumor growth in individual mice treated as indicated. A complete response (CR) was defined as no detectable tumor mass at day 150, and partial response (PR) was defined as a tumor that exhibited no change, or a reduction, in size for 50 days, followed by remission. N = 5 for PBS+αPD-1 and IR+PBS+αPD-1 treatment groups, and N = 8 for the rest. Panel C. Kaplan-Meier plot comparing survival associated with each treatment regimen. Also shown are the median survival (MS) times. P values determine from Log-rank (Mantel-Cox) testing comparing individual growth curves.

The highest levels of PD-1 expression on CD4^+^ and CD8^+^ cells were most often seen in mice treated with VACV alone or with VACV in combination with IR (Fig 6A–6D). Conversely, IR treatment increased the proportion of PD-L1 on the non-hematopoietic cells recovered from the treated tumors and the effect was also seen when IR was combined with VACV (Fig 6E). Collectively, this would create an unfavorable situation where VACV exposure is promoting development of CD8^+^ T-cells exhibiting a marker characteristic of T-cell exhaustion (the T_EX_ state, [34]), while at the same time the IG-IR exposure has upregulated inhibitory ligands on the surface of surviving tumor cells. This led us to postulate that the antagonism between radiation and oncolytic VACV might be reversed by checkpoint inhibition of T-cells. To test this, we performed a final experiment testing a triple-combination therapy of radiation, virus and αPD-1 antibody (Fig 7A).

However, despite the increases in PD-1 and PD-L1, adding αPD-1 checkpoint therapy to the treatment regimen did not reverse the antagonism (Fig 7B and 7C). When we compared IR+VACV+αPD-1 with IR+VACV+isotype (Fig 7C), we saw no difference in the outcomes suggesting the hypothesis was wrong. However, that interpretation is complicated by the control arms in the study, in particular the observations that the IR+PBS+αPD-1 and PBS+αPD-1 treatments also yielded fewer survivors than the IR+PBS+isotype treatment. (What caused this peculiar effect is not clear although it has been observed that PD-1 or CTLA-4 blockade can promote other effects including tumor vascular normalization [35]. This would have uncertain consequences in TUBO tumors.) Whatever the reason(s), these data provide no clear evidence to support the hypothesis that immune cell exhaustion is contributing to the antagonism observed in tumor clearance. Another mechanism must be responsible for the negative interplay between radiation and oncolytic virotherapy in this model.

## Discussion

This study was designed to test if an oncolytic VACV could be combined with IG-IR, to improve treatment response of an orthotopic tumor in an immune competent host. It was prompted by prior research showing that IR can synergize under certain circumstances with oncolytic VACV to improve the therapeutic outcome [19, 21, 36–39]. It is difficult to compare our results with many of these studies given the differences in how the radiation was delivered, the viruses, the tumor models, and the immunological status of the treated animals. Xenografted tumors, for example, are highly susceptible to oncolytic virotherapy, but offer few insights into the challenge of treating tumors in immune-competent hosts. However, we have recently published a study showing that a ΔF4LΔJ2R VACV strain can synergize with IG-IR in an orthotopic and immune-competent murine glioblastoma model, to improve the cure rate 3-to-4-fold relative to either monotherapy [22]. Chen *et al*. also showed that a combination of VACV plus stereotactic body radiation extended the survival of immune competent mice bearing TC-1 E6^+^E7^+^*ras*^+^ lung epithelial cell derived tumors relative to monotherapies alone [39]. Thus, the approach can work under some circumstances.

Solid tumors generally respond poorly to virotherapy alone, and this study also tested whether debulking a tumor with radiation could provide more time for VACV to effect a better anti-tumor response. For this to work it was assumed that VACV could infect and kill irradiated tumor cells, while hopefully enhancing an immunological response to any cells that have survived both insults. A particular advantage of IG-IR technology is that it targets the radiation to the tumor site while limiting damage in nearby lymphoid organs. This also provided an opportunity to explore what role a reservoir of splenic lymphocytes might play in modulating tumor growth following tumor treatment with IR and/or ΔF4LΔJ2R VACV.

This study showed that ΔF4LΔJ2R VACV grows well in irradiated breast cancer cells and that IR can synergize with VACV to kill TUBO and 4T1 cells *in vitro* (Fig 1). Nevertheless, the results obtained with an orthotopic glioblastoma model [22] were not replicated in breast cancer models. VACV therapy alone was the least effective TUBO tumor treatment tested, producing a worse outcome than what was seen in the PBS-treated controls (Fig 2). Combining VACV with IG-IR improved the response by delaying the disease progression and increasing overall survival. However, combining these two treatments was not as effective as IG-IR alone; virus treatment arguably antagonized the response to radiation. A somewhat different effect was seen in a metastatic 4T1 breast cancer model. VACV alone had no effect compared to PBS-treated controls, and although VACV plus IG-IR produced an improved response compared to virus monotherapy, IR alone was still as good or better (Fig 2D). VACV antagonizes TUBO tumor treatments and has no effect on 4T1 treatment. Why does combining IR and VACV not improve breast cancer therapy?

From an immunological perspective, a contradictory feature of treating TUBO tumors with VACV monotherapy is that the properties of the TILs retrieved from the TME seem to align well with what is expected for a promising prognosis. Monotherapy caused an expansion in the numbers of CD4^+^ and CD8^+^ T cells and an associated increase in the CD8^+^/T_reg_ ratio (Fig 4). This population of CD8^+^ T cells comprises cells exhibiting CD69^+^ activation markers and recognizing both VACV A52_75-83_ and tumor p66 HER/neu epitopes (Fig 5). Nevertheless, VACV treatments alone provided little benefit. An increased percentage of PD-1^+^CD8^+^ T cells was detected in VACV-treated tumors (Fig 6), but PD-1 can be an activation marker and its presence is not necessarily evidence that a T-cell population has progressed to a terminally exhausted state [34]. Certainly αPD-1 checkpoint inhibition did not improve the situation (Fig 7). By comparison, tumors that were treated with IR alone exhibited a large reduction in the numbers of TILs of any type (Fig 4), with almost undetectable numbers of p66-HER2/*neu* tetramer positive CD8^+^ T cells (Fig 5). Despite this depletion of CD8^+^ T cells, the tumors were still eventually eradicated in many more of the animals in the “only IR” cohort. These survivors were all subsequently challenged with fresh TUBO cells and nearly all (9 of 10) still exhibited an anti-tumor response characteristic of acquired cellular immunity (Fig 5F). p66-HER2/*neu* tetramer positive CD8^+^ T cells were detected in the spleens of IR-treated animals (Fig 5C), which may have served as a refugia for the CD8^+^ T cells that were depleted from the tumors by IR.

The TILs recovered from TUBO tumors treated with both VACV and IR generally exhibited hybrid properties (e.g., Fig 4B and 4C) although one notable difference was that the numbers of inhibitory cell types (T_reg_, M-MDSC, and PMN-MDSC) resembled the low levels seen in IR-treated tumors compared to the high levels detected in VACV-treated tumors (Fig 4D–4F).

These effects appear to be time dependent as delaying the VACV treatment for three weeks after the IR eliminated the antagonism, albeit yielding outcomes no different from IR alone. In this case TUBO tumor development paused and some tumors shrank following IR plus virotherapy (Fig 3A), presumably due to viral oncolysis. This treatment regimen would have also benefited from the short-term impact of IR through direct killing, supplemented by any immunogenic cell death and enhanced by an anti-tumor response. It would have been aided by the aforementioned recruitment of p66-HER2/*neu* reactive CD8^+^ T cells from an intact splenic reservoir. Collectively the two effects (oncolysis plus T cell recruitment) could extend survival but ultimately both still failed to clear the tumors any better than IR alone.

We did not investigate immune responses in 4T1 tumors so exhaustively. The most interesting thing about these data was that tumors extracted from VACV-treated animals exhibited an immunological profile closely resembling the immune response detected in PBS-treated controls. Likewise, the types and numbers of TILs detected in tumors treated with VACV plus IG-IR looked like IR-treated tumors (S1 Fig). The fact that VACV treatment either alone or combined with IR, had no effect on survival in these mice, is fully consistent with the fact that virus treatment had no obvious impact on the composition of the TME.

The most striking difference between these data and the immune analyses we reported previously [22] is that PBS-treated (i.e., control) glioblastomas entrain few CD8^+^ T cells of any specificity and this low pre-existing background changed little in response to IR alone. ΔF4LΔJ2R VACV treatment dramatically elevated the levels of CD4^+^ and CD8^+^ cells and adding IG-IR therapy further increased the CD8^+^/T_reg_ ratio [22]. In contrast one can recover an abundance of CD8^+^ T cells from PBS-treated TUBO tumors and spleens (Fig 4) and this background included some p66 HER2/*neu* reactive T cells (Fig 5). VACV treatment further enhanced these numbers, but the continued growth of these tumors despite the pre-existing or boosted cytotoxic responses, suggests that the TUBO TME comprises an environment far less susceptible to immunotherapy than CT2A glioblastomas.

Trying to rationalize these observations is a challenge and it may be that that there are idiosyncratic features of TUBO tumor models that preclude extending the observations to solid-tumor treatments in general. In summary, what we see is that when TUBO tumors are treated with IR plus VACV, it alters the composition of TILs and these effects are superficially positive in nature. Most notably, virus exposure promoted the recruitment of CD8^+^ T cells, including p66 HER2/*neu* tetramer positive T cells, to a tumor environment depleted of these populations by IR (Figs 4 and 5). Although these T cells also exhibit increased levels of PD-1, and the irradiated tumor cells expressed upregulated PD-L1^+^ (Fig 6), the simplest interpretation of an experiment involving the use of a αPD-1 inhibitor is that it still provided no improvement (Fig 7).

At present, the most likely explanation for what is causing this effect relates to the observation that it is time dependent and dissipates if VACV exposure is delayed for three weeks (Fig 3). Orthopoxviruses like VACV encode a remarkable number of genes capable of inhibiting elements of both the innate and adaptive immune systems [40] including type I/II IFN and NFKB signaling [41, 42] and effector T-cell functions (e.g., [43]). Several apoptotic inhibitors are encoded by these viruses [44]. Most notably VACV B2R encodes a cGAMP nuclease [45] and E5R was also recently shown to encode an inhibitor of the DNA sensor cGAS [46]. The cGAS-STING pathway provides an important link between radiation and the immune response to radiation. It is slowly triggered (over days) as irradiated cells transit through the cell cycle, releasing DNA to be sensed by cGAS [47] and promotes many responses linked to Type I IFN signaling including DC priming of CD8^+^ T cells (reviewed in [48]). Given the substantive array of immunosuppressive VACV-encoded gene products, the most parsimonious explanation is that by delivering the virus so soon after the exposure to IR, we are interfering in both cell killing and an innate immune response that is required for radiotherapy to achieve a beneficial impact.

Going forward many new lines of investigation are suggested by this study. Ideally, it would be informative to test whether administering the virus prior to radiation would yield a different effect, but for now that study has been precluded by biosafety constraints in our research facility. Such an approach might better deploy the increased numbers of p66-HER2/*neu* reactive CD8^+^ T cells that are detected in virus-infected animals (Fig 5D) and would not be expected to interfere in the pro-immunogenic effects of IR if timed correctly. Fractionated doses of radiation can also be delivered using a SARRP and are known to be more immunogenic than single-dose IR [49, 50]. Most importantly, there is a need to minimize the immune suppression promoted by a full VACV gene complement. The two most obvious targets would be E5R and B2R along with VACV genes that inhibit IFN signaling (B8R and B18R). Several of these gene knockouts are known to improve the efficacy of oncolytic VACV-immunotherapy [14, 51, 52]. VACV also encodes systems potentially able to inhibit radiation induced apoptosis [44] and another soluble gene product (M2) that blocks the action of the co-stimulatory molecules CD80 and CD86 [43]. Many of these different VACV mutants would likely work better when combined with IG-IR.

In conclusion, these results show that more is not necessarily better when one combines therapies in cancer treatment. While many reports show that IR can synergize with viruses to enhance killing of cancer cells, our observations show that *in vitro* assays do not always predict the outcomes in an *in vivo* context especially in an immune-competent host. Considering the potential risk this poses, more studies are needed to fully understand how these two therapies interact *in vivo* to produce the effects we’ve seen. Such studies would also be expected to produce important insights into how one might improve the effectiveness of both IR and oncolytic viruses.

## Materials and methods

### Cell lines and culture conditions

TUBO mouse mammary carcinoma cells were kindly provided by Dr. L. Landuzzi (University of Turin) [53]. Charles River Laboratories (Wilmington, MA) documented the cell line identity and freedom from adventitious agents. 4T1, MDA-MB-231, and MCF7 cells were purchased from the ATCC, while MTHJ cells [54] were provided by Dr. K. Mossman (McMaster University). Breast cancer cells were cultured in Dulbecco’s modified Eagle’s medium (DMEM) containing 2mM L-glutamine, 100U/mL anti-mycotic/antibiotic, non-essential amino acids and 1mM sodium pyruvate supplemented with 20% fetal bovine serum (FBS) for TUBO cells or 10% FBS for the remaining cells. BSC-40 cells were purchased from ATCC in 2005 and grown in minimal essential medium supplemented as above with 5% FetalGro bovine growth serum (RMBIO). All cells were passaged <20 weeks, and regularly mycoplasma tested.

### Virus stocks and plaque assay

ΔF4LΔJ2R VACV was derived from VACV strain Western Reserve (WR) [12–14, 55]. PCR and whole-genome sequencing were used to confirm the identity of the recombinant virus. Virus stocks were prepared as described [14]. Briefly, BSC-40 cells were infected for 3 days, harvested with a cell scraper in hypotonic buffer, and broken by Dounce homogenization. Extracellular nucleic acids were digested with Benzonase, the stock purified by centrifugation through a 36% sucrose cushion, the virus titered on BSC-40 cells, and aliquoted for storage at -80˚C. After thawing the virus in preparation for tumor treatments, a portion was put aside and retitered by plaque assay.

For plaque assays, virus samples were diluted in PBS and 300 μL volumes plated in triplicate on BSC-40 cells in 12-well plates. The dishes were incubated for 1 hr with rocking at 37°C and then the inoculum was replaced with fresh media containing 1% carboxymethylcellulose. After 2 days of culture at 37˚C, the cells were fixed and stained. The infectious virus titer was determined from the plaque counts.

### Cell irradiation and virus growth curves

Cells were transported to the Cross Cancer Institute (CCI) in an insulated container, 24 hr post-seeding in 6-well plates. The cells were irradiated using a GammaCell irradiator and then returned to the university where they were cultured at 37°C until further treatment. For multistep growth curves, the cells that had been irradiated 24 hr previously were infected with VACV at a multiplicity of infection (MOI) of 0.03 PFU/cell for 1 hr. The inoculum was then removed and replaced with fresh media. The cells were harvested with a cell scraper at different time points post infection and freeze-thawed three times at -80° C. The virus titers were determined by plaque assay as described above.

### Cytotoxicity assays and synergy analysis

Cells were seeded in 96‐well plates, irradiated (or not), and 24 hr later infected with VACV at different MOI’s in triplicate wells. The medium was replaced 3 days later with fresh culture medium containing 44 μM resazurin (Sigma‐Aldrich). The plates were incubated for 4 hr at 37°C, and fluorescence was measured using a microplate reader with 560‐nm excitation and 590‐nm emission filters. Cell survival was expressed as a percentage, based on the fluorescence of untreated mock-infected cells (100% survival) and after subtracting the background detected in cells killed with 10% Triton X-100 (0% survival).

CombuSyn software (http://www.combosyn.com/) was used to determine combination index (CI) values using data obtained from resazurin assays [24]. The three experimental replicates were used to calculate an “average value of effect size” defined as the fraction of dead cells following treatment. Interactions were assessed using the “non-constant” drug ratio setting. The CI values and Prism software were used to generate the heatmaps using the scale shown in S1 Table. Because none of the computed CI values were >1.88, the heatmap scale was clipped at CI = 3, representative of “antagonism” [24, 25].

### Tumor models

Animal studies were carried out according to Canadian Council on Animal Care Guidelines and Policies (https://ccac.ca/en/guidelines-and-policies/). Approvals were provided by the CCI Animal Care Committee and by the University of Alberta Health Sciences Animal Care and Use Committee (animal use protocol AUP00001914).

Six-to-eight-week-old female BALB/c mice were purchased from Charles River (Saint Constant, Québec). Animals were received and housed at the CCI animal facility and given at least 7 days to acclimate after arrival, or (following irradiation) housed at the University of Alberta BSL-2 animal facility and given 24 hr to acclimate. Animals were housed in groups of four or five in negatively ventilated cages (Animal Care Systems, CO) at the CCI, or HEPA-filtered ventilated cages (Ehret, Germany) at the University of Alberta with environmental enrichments. Food (LabDiet, St. Louis, MO) and water were provided *ad libitum*.

To establish orthotopic tumors the cells were trypsinized, washed twice with cold PBS, and concentrated by centrifugation at 500×G for 5 min. The cells were kept on ice during processing. Immediately prior to injection, 25 μL of Matrigel was mixed with 25 μL of cells to yield a dose of 10^6^ TUBO cells or 10^4^ 4T1 cells per mouse. Animals were anaesthetized with isoflurane, and the cells injected into the inguinal mammary fat pad below the fourth nipple. Palpable tumors were detected after about 8 days in both TUBO and 4T1 tumor models. The same procedure was followed for tumor re-challenge studies, except the cells were injected into the mammary fat pad opposite where the first tumor had been. To track tumor development, the animals were anaesthetized, weighed, and tumor growth was measured with calipers. Tumor volume was calculated using the equation V = (1/24)×π×L×(W+H)^2^. Mice were euthanized with CO_2_ once the tumor burden reached 1,500 mm^3^ or at first indication of illness or discomfort (hunched posture, ruffled fur, or weight loss >10% of body weight).

### Oncolytic virus administration

A random number generator was used to assign mice to treatment groups. Viruses were sonicated in a cup horn sonicator for 90 sec to dissociate aggregates and diluted to 2×10^8^ PFU/mL with PBS. Mice were anaesthetized with isoflurane and injected intratumorally with 50 μL of virus delivering 10^7^ PFU/tumor. Animals received two more doses at 48 hr intervals. Where virus treatment was supplemented with checkpoint inhibition, the mice were anaesthetized and 200 μg of αPD-1 antibody (Clone RMP1-14, Bio X Cell, New Hampshire USA) or an isotype control were administered intraperitoneally twice per week for three weeks.

### SARRP treatment

Mice were treated with IG-IR once palpable tumors were detected. Mice were anaesthetized with isoflurane and transferred to the stage of a small animal radiation research platform instrument (SARRP; XStrahl Inc, Sunwanee, GA). Computed tomography (CT) images were acquired and uploaded into the XStrahl MuriPlan treatment planning software. During treatment planning, a heat lamp was placed near the stage, and an operator continually monitored the anesthetized mouse. The CT images plus manual and automatic tissue segmentation were used to adjust the radiation path. The tumor image was contoured from DICOM slices, and that model used to register its size and location and compute the isocenter where the X-ray beams converge. The angles and weighted dose of each beam were adjusted for each mouse to limit radiation exposure to non-tumor tissue, especially bone. Each isocenter received 10 Gy, split between beams, with each beam delivering 20–50% of the total dose. MuriPlan software was used to ensure that the planned treatment would deliver ≥90% of a 10 Gy dose to ≥80% of the tumor. The mice were returned to their cages and subsequently transported to the University of Alberta for virus treatment.

### Immunological analysis

Splenocytes were isolated as described [7]. Briefly, spleens were mashed through a 70 μm strainer into isolation buffer containing 2% heat-inactivated FBS (HI-FBS) and 0.5 mM EDTA in PBS. The cells were centrifuged at 300 x G for 5 min and resuspended in 5 mL red blood cell lysis buffer (eBioscience). Lysis was stopped by adding 10 mL isolation buffer, the splenocytes were recovered by centrifugation 300 × G for 5 min, washed twice with isolation buffer, and live cell counts determined using an automated cell counter (Life Technologies, Carlsbad CA, USA).

Tumor processing was performed as described [14]. Briefly, tumors were collected in Hank’s buffered salt solution (HBSS) and minced with a scalpel. The pieces were dissociated using the “m_impTumor01.01” protocol on a GentleMACs dissociator (Miltenyi Biotec) in 5mL of Roswell-Park Memorial Institute (RPMI) medium containing 0.5 mg/mL collagenase (Sigma-Aldrich), 10 μg/mL DNAse I (Roche) and 10% HI-FBS. The sample was incubated for 30 min at 37°C, filtered through a 70 μm strainer into 2% HI-FBS in PBS and concentrated at 500 × G for 5 min. The cells were resuspended in 40% Percoll (GE Healthcare) in HBSS, layered over 80% Percoll and centrifuged at 325 × G for 30 min. The leukocytes were recovered from the interface.

For flow cytometry, 2×10^6^ splenocytes were aliquoted into 96-well plates, and the remainder were pooled for use as fluorescence minus one (FMO) gating controls. For tumor samples, all the tumor cells were split between 1 (4T1) or 2 wells (TUBO) of a 96-well plate with 5% of each sample volume reserved as control wells. The cells were rinsed in PBS, and stained with eflour506 viability dye (Invitrogen, Cat. 65–086614). This and subsequent staining steps, were performed in the dark for 30 min at 4°C. Fc receptors were blocked using anti-CD16/CD32 antibody (BioLegend). The cells were stained with the antibodies listed in S2 Table at a dilution of 1:200 and then fixed and permeabilized using a BD Cytofix/Cytoperm kit (San Jose, CA, USA). Data was acquired using a BD Fortessa X20 flow cytometer and analyzed with FlowJo v8 or v10 software. All the tumor sample was run to measure absolute cell counts. An example of the gating strategy is shown in S3 Fig.

### Statistical analysis

Data were analyzed using GraphPad Prism 7. If data were determined to be normally distributed by the Shapiro-Wilk normality and the Kolmogororv-Smirnov tests, parametric one-way ANOVA testing was performed with Tukey’s multiple comparisons tests. If data were not parametric, Kruskal-Wallis testing was performed with Dunn’s multiple comparisons tests. Significance was determined if p ≤ 0.05. Tumor growth curves were analyzed using a two-way ANOVA. Survival data were analyzed by log-rank (Mantel-Cox) testing.

## Supporting information

S1 Fig 4T1 tumor model. Combining radiation and VACV therapy changes the 4T1 tumor and splenic immune cell microenvironment. Tumors were established and treated as in Fig 2A, and the tissues retrieved and processed one week after the last virus (or mock virus) treatment. Upper panel, total number of each immune cell type in 4T1 tumors: (A) CD3^+^ T cells, (B) CD4^+^ T cells, (C) CD8^+^ T cells, (D), T_reg_ cells, (E) M-MDSC cells, and (F) PMN-MDSC cells. Lower panel, average composition of immune cells in the spleens of mice harbouring 4T1 tumors as a percentage of CD45^+^ cells. Tumor-free mice are shown for a comparison. (G) CD3^+^ T cells, (H) CD4^+^ T cells, (I) CD8^+^ T cells, (J), T_reg_ cells, (K) M-MDSC cells, and (L) PMN-MDSC cells. The averages were calculated using 5 mice per treatment group, 4 if tumor free. Error bars depict 95% CI from the mean, where *p<0.05, **p<0.01, ***p<0.005 and ****p<0.001.
(TIF)

S2 Fig Virus titers are decreased in irradiated tumors one week after virus therapy. Tumors were established, irradiated, treated with ΔF4LΔJ2R VACV, and the mice euthanized one week following the final virus treatment. Tumors were collected, dissociated by enzymatic digestion, and the tumor cells were separated from immune cells on a Percol gradient. The infectious virus isolated from **A** TuBo and **B** 4T1 tumors were quantified by plaque assay on BSC-40 cells. Each symbol represents an individual mouse. *n* = 5 mice per group. P values reported using Mann-Whitney testing.
(TIF)

S3 Fig Representative flow cytometry gating strategy used to identify immune cell subsets and marker expression on splenic and tumor cells. A fluorescence minus one (FMO) gating control was used to set the gates.
(TIF)

S1 Table Synergy measurements. Interpretation of CI values for levels of synergy and antagonism. Table adapted from Chou, 2006.
(DOCX)

S2 Table Flow cytometry reagents. (DOCX)

S1 Questionnaire Inclusivity in global research questionnaire. (DOCX)
