# Supplementary figures and images for: Oncolytic vaccinia virus immunotherapy antagonizes image-guided radiotherapy in mouse mammary tumor models
Source: PLoS One. 2024 Mar 18;19(3):e0298437. doi: 10.1371/journal.pone.0298437 (PMC10947714; doi:10.1371/journal.pone.0298437)

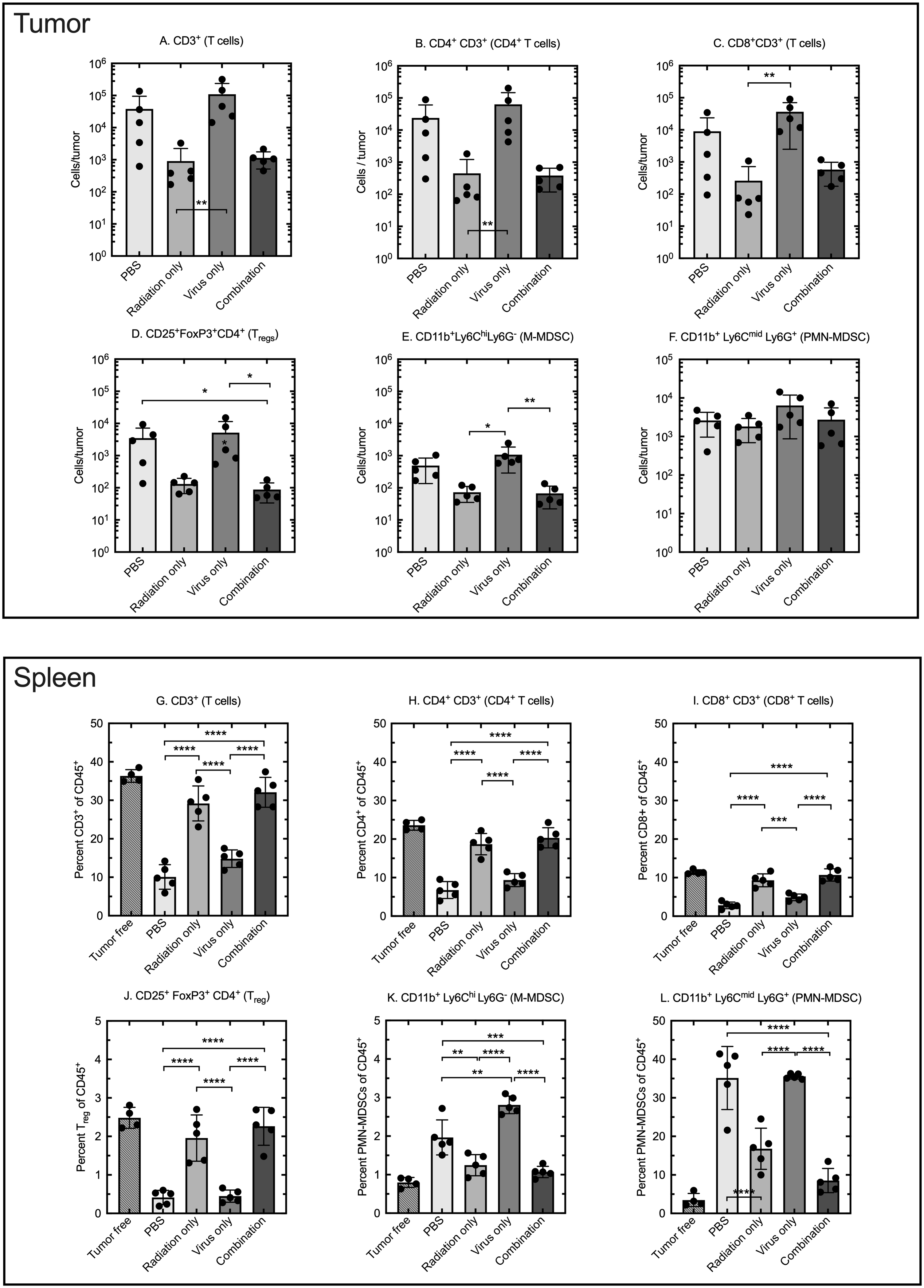

Supplement: S1 Fig — Combining radiation and VACV therapy changes the 4T1 tumor and splenic immune cell microenvironment. Tumors were established and treated as in Fig 2A, and the tissues retrieved and processed one week after the last virus (or mock virus) treatment. Upper panel, total number of each immune cell type in 4T1 tumors: (A) CD3+ T cells, (B) CD4+ T cells, (C) CD8+ T cells, (D), Treg cells, (E) M-MDSC cells, and (F) PMN-MDSC cells. Lower panel, average composition of immune cells in the spleens of mice harbouring 4T1 tumors as a percentage of CD45+ cells. Tumor-free mice are shown for a comparison. (G) CD3+ T cells, (H) CD4+ T cells, (I) CD8+ T cells, (J), Treg cells, (K) M-MDSC cells, and (L) PMN-MDSC cells. The averages were calculated using 5 mice per treatment group, 4 if tumor free. Error bars depict 95% CI from the mean, where *p<0.05, **p<0.01, ***p<0.005 and ****p<0.001. (TIF) [file pone.0298437.s001.tif]

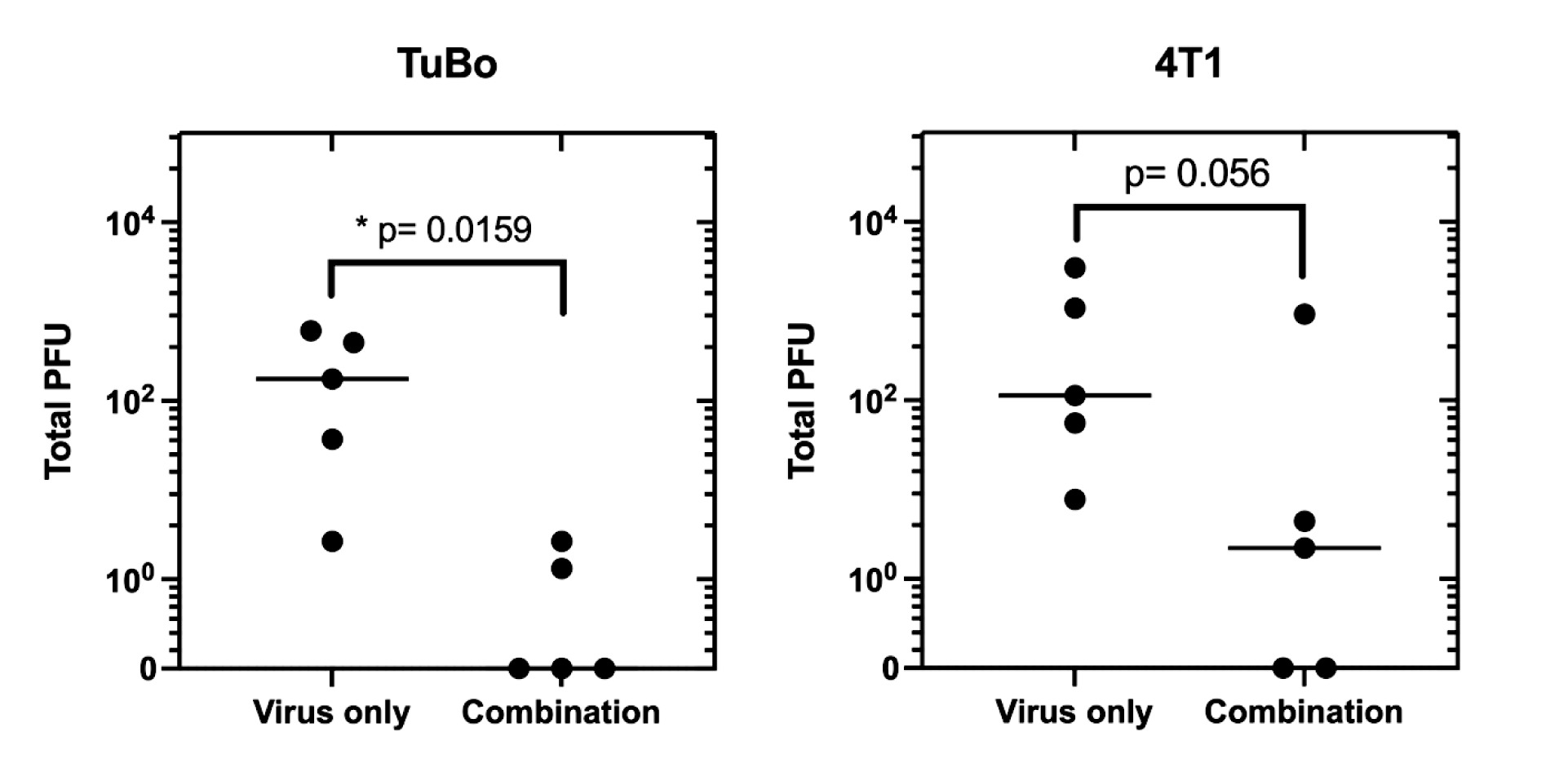

Supplement: S2 Fig — Tumors were established, irradiated, treated with ΔF4LΔJ2R VACV, and the mice euthanized one week following the final virus treatment. Tumors were collected, dissociated by enzymatic digestion, and the tumor cells were separated from immune cells on a Percol gradient. The infectious virus isolated from A TuBo and B 4T1 tumors were quantified by plaque assay on BSC-40 cells. Each symbol represents an individual mouse. n = 5 mice per group. P values reported using Mann-Whitney testing. (TIF) [file pone.0298437.s002.tif]

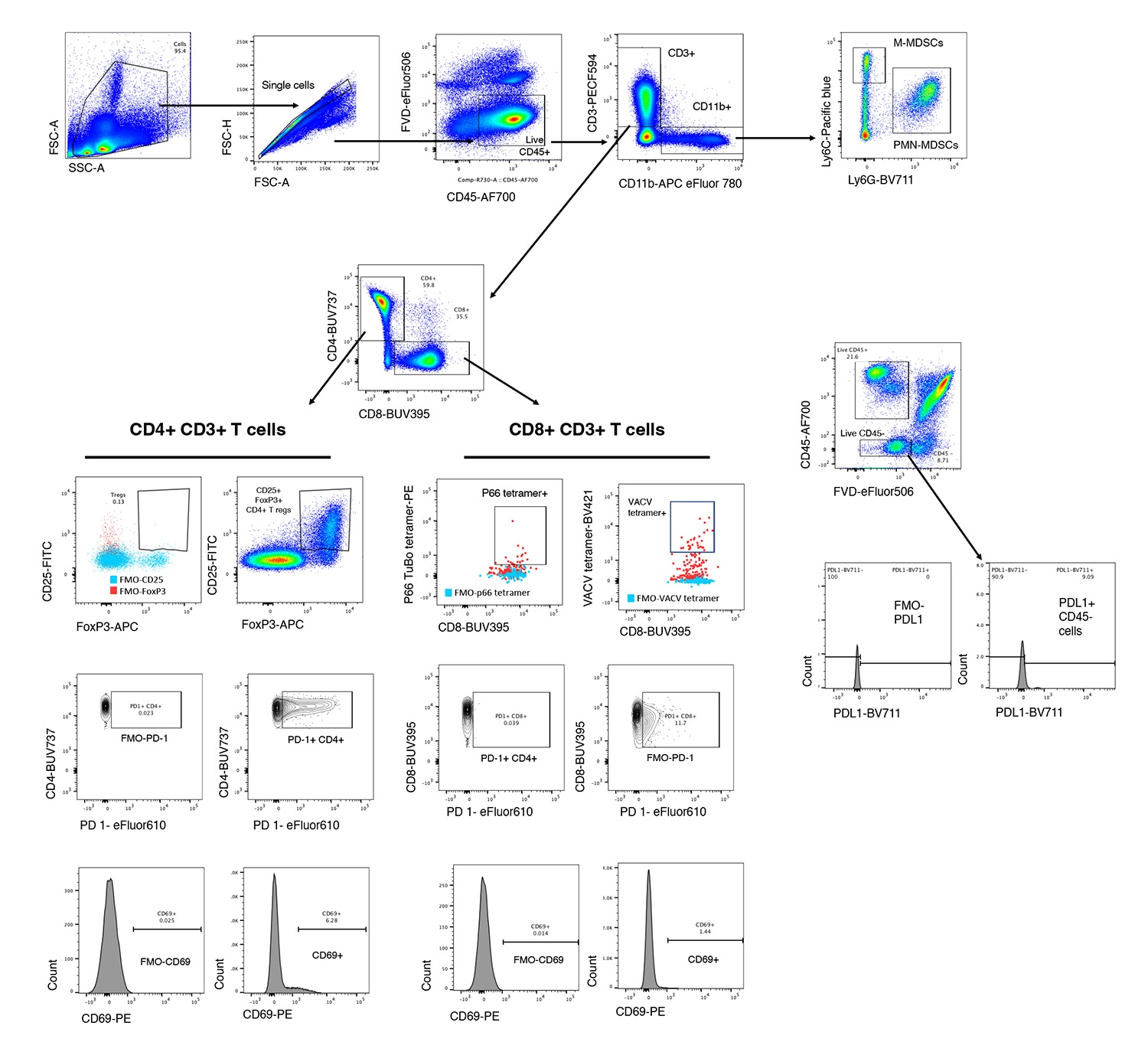

Supplement: S3 Fig — A fluorescence minus one (FMO) gating control was used to set the gates. (TIF) [file pone.0298437.s003.tif]
