# Supplementary material for: Oncolytic vaccinia virus immunotherapy antagonizes image-guided radiotherapy in mouse mammary tumor models
Source: PLoS One. 2024 Mar 18;19(3):e0298437. doi: 10.1371/journal.pone.0298437 (PMC10947714; doi:10.1371/journal.pone.0298437)
Supplement: S1 Table — Interpretation of CI values for levels of synergy and antagonism. Table adapted from Chou, 2006. (DOCX) [file pone.0298437.s004.docx]

**Table S1**. Interpretation of CI values for levels of synergy and antagonism. Table adapted from Chou, 2006.

| **CI value** | **Interpretation** |
| --- | --- |
| <0.1 | Very strong synergism |
| 0.1-0.3 | Strong synergism |
| 0.3-0.7 | Synergism |
| 0.7-0.85 | Moderate synergism |
| 0.85-0.9 | Slight synergism |
| 0.9-1.1 | Nearly additive |
| 1.1-1.20 | Slight antagonism |
| 1.20-1.45 | Moderate antagonism |
| 1.45-3.3 | Antagonism |
| 3.3-10 | Strong antagonism |
| >10 | Very strong antagonism |
