# Supplementary material for: Oncolytic vaccinia virus immunotherapy antagonizes image-guided radiotherapy in mouse mammary tumor models
Source: PLoS One. 2024 Mar 18;19(3):e0298437. doi: 10.1371/journal.pone.0298437 (PMC10947714; doi:10.1371/journal.pone.0298437)
Supplement: S2 Table — (DOCX) [file pone.0298437.s005.docx]

| **Antibody or reagent** | **Clone** | **Source** |
| --- | --- | --- |
| CD45-AF700 | 30-F11 | Invitrogen |
| CD3-PE or FITC or PE-CF594 | 145-2C11 | Invitrogen or BD Bioscience |
| CD4-BUV737 | RM4-5 | BD Bioscience |
| CD8-BUV395 | 53-6.7 | BD Bioscience |
| CD25-FITC | P4A10 | Invitrogen |
| FoxP3-AF647 | FJK-16S | Invitrogen |
| CD69-APCefluor780 or PE | H1.2F3 | Invitrogen |
| CD11b-APCefluor780 | M1/70 | Invitrogen |
| Ly6C-efluor450 | HK1.4 | Invitrogen |
| Ly6G-biotin | IA8 | Biolegend |
| PD1-efluor610 | J43 | Invitrogen |
| PDL1-biotin | 10F.9G2 | Biolegend |
| Streptavidin-BV711 | Not applicable | BD Bioscience |
| H-2K^d^ VACV A52_75-83_ KYGRLFNEI- BV421 Tetramer | Not applicable | NIH Tetramer Facility |
| H-2K^d^ HER2/*neu* p66 TYVPANASL- PE Tetramer | Not applicable | NIH Tetramer Facility |
